# Neurological disorders affecting the lower limb in children

**DOI:** 10.1186/1757-1146-4-S1-I10

**Published:** 2011-05-20

**Authors:** Monique Ryan

**Affiliations:** 1 Children’s Neurosciences Centre, Royal Children’s Hospital, Melbourne 3052, Australia; 2 Department of Paediatrics, University of Melbourne, Victoria, Australia; 3 Murdoch Childrens Research Institute, Melbourne, Australia

## 

Changes in foot and ankle posture are a normal aspect of infant and child development but may also reflect neurological conditions affecting the brain, spinal cord, peripheral nerves or muscles. Development of cavus or planus foot deformities often reflects underlying neurologic disorders and may predate development of more overt neurological signs. This session will concentrate upon signs and symptoms suggestive of occult neurologic problems in children presenting with positional or developmental abnormalities of the lower limbs.

